# Real-world evidence of the effectiveness of ombitasvir-paritaprevir/r ± dasabuvir ± ribavirin in patients monoinfected with chronic hepatitis C or coinfected with human immunodeficiency virus-1 in Spain

**DOI:** 10.1371/journal.pone.0225061

**Published:** 2019-11-12

**Authors:** José Manuel Sousa, Mercedes Vergara, Federico Pulido, Gloria Sánchez Antolín, Lander Hijona, Fernando Carnicer, Diego Rincón, Javier Salmerón, Beatriz Mateos-Muñoz, Antoni Jou, Benjamín Polo-Lorduy, Ángel Rubín, Ana Escarda, Patricia Aguilar, Teresa Aldámiz-Echevarría, Luisa García-Buey, José A. Carrión, Manuel Hernández-Guerra, Sonia Chimeno-Hernández, Nuria Espinosa, Rosa Mª Morillas, Raúl J. Andrade, Manuel Delgado, Adolfo Gallego, Marta Magaz, José María Moreno-Planas, Ángel Estébanez, Mikel Rico, Fernando Menéndez, Blanca Sampedro, Luís Morano, Sonia Izquierdo, José Manuel Zozaya, Manuel Rodríguez, Senador Morán-Sánchez, Sara Lorente, Ignacio Martín-Granizo, Miguel Ángel Von-Wichmann, Marcial Delgado, Amanda Manzanares

**Affiliations:** 1 Hepatology Department, Hospital Universitario Virgen del Rocío, Sevilla, Sevilla, Spain; 2 Hepatology Unit, Digestive Disease Department, Parc Taulí Sabadell Hospital Universitari, Sabadell, Barcelona, Spain, CIBERehd, Instituto Carlos III, Madrid, Spain; 3 HIV Unit, Hospital Universitario 12 de Octubre, imas12, Universidad Complutense de Madrid (UCM), Madrid, Spain; 4 Hepatology Department, Hospital Universitario Río Hortega, Valladolid, Valladolid, Spain; 5 Hepatology Department, Hospital Universitario Araba, Vitoria-Gasteiz, Álava, Spain; 6 Hepatology Department, Hospital General Universitario de Alicante, Alicante, Alicante, Spain; 7 Hepatology Department, Hospital General Universitario Gregorio Marañón, CIBERehd and (UCM), Madrid, Spain; 8 Hepatology Department, Complejo Hospitalario Universitario de Granada, Granada, Granada, Spain; 9 Hepatology Department, Hospital Universitario Ramón y Cajal, Madrid, Spain; 10 HIV Clinical Unit, Internal Medicine Department and Fundació de la Lluita contra la SIDA, Hospital Universitari Germans Trias i Pujol, Universitat Autònoma de Barcelona, Barcelona, Spain; 11 Hepatology Department, Hospital Universitario Fundación Jiménez Díaz, Madrid, Spain; 12 Hepatology Department, Hospital Universitario y Politécnico de La Fe, Valencia, Valencia, Spain; 13 Hepatology Department, Hospital Universitario Son Espases, Palma de Mallorca, Spain; 14 Digestive System Clinical Unit, Instituto Maimónides de Investigación Biomédica de Córdoba (IMIBIC), Hospital Universitario Reina Sofía/Córdoba University, Córdoba, Spain; 15 Infectious Diseases-HIV, Hospital General Universitario Gregorio Marañón (IiSGM), Madrid, Madrid, Spain; 16 Hepatology Department, Hospital Universitario de La Princesa, Madrid, Spain; 17 Liver Section, Gastroenterology Department, Hospital del Mar, IMIM (Hospital del Mar Medical Research Institute), UAB (Universitat Autonoma de Barcelona) Barcelona, Spain; 18 Hepatology Department, Hospital Universitario de Canarias, La Laguna, Santa Cruz de Tenerife, Spain; 19 Hepatology Department, Hospital Arnau de Vilanova, Valencia, Valencia, Spain; 20 Clinical Unit of Infectious Diseases, Microbiology and Preventive Medicine, Infectious Diseases Research Group, Institute of Biomedicine of Seville (IBiS), University of Seville/CSIC,Sevilla, Spain; 21 Liver Section, Department of Gastroenterology, Hospital Universitari Germans Trias i Pujol, IGTP, Badalona, Barcelona, Spain, and CIBEREHD; 22 Unidad de Gestión Clínica de Aparato Digestivo, Instituto de Investigación Biomédica de Málaga-IBIMA, Hospital Universitario Virgen de la Victoria, Universidad de Málaga, Centro de Investigación Biomédica en Red de Enfermedades Hepáticas y Digestivas CIBERehd, Málaga, Spain; 23 Hepatology Department, Complejo Hospitalario Universitario A Coruña, A Coruña, Spain; 24 Hepatology Department, Hospital de la Santa Creu i Sant Pau, Barcelona, Barcelona, Spain; 25 Hepatology Department, Hospital Universitario Puerta de Hierro Majadahonda, Madrid, Spain; 26 Digestive System Department, Complejo Hospitalario Universitario de Albacete, Albacete, Spain; 27 Hepatology Department, Hospital Universitario Marqués de Valdecilla, Santander, Cantabria, Spain; 28 Infectious Diseases Unit, Hospital Universitario La Paz, Madrid, Spain; 29 Hepatology Department, Hospital Universitario Basurto, Bilbao, Vizcaya, Spain; 30 Hepatology Department, Hospital Galdakao, Galdakao, Vizcaya, Spain; 31 Infectious Diseases Unit, Hospital Universitario Álvaro Cunqueiro, Vigo, Pontevedra, Spain; 32 Hepatology Department, Hospital Clínico San Carlos, Madrid, Spain; 33 Hepatology Department, Complejo Hospitalario de Navarra, Pamplona, Navarra, Spain; 34 Liver Unit, Division of Gastroenterology & Hepatology. Hospital Universitario Central de Asturias, Oviedo, Asturias, Spain; 35 Hepatology Department, Hospital General Universitario Santa Lucía, Cartagena, Murcia, Spain; 36 Hepatology Department, Hospital Clínico Universitario Lozano Blesa, Zaragoza, Zaragoza, Spain; 37 Department of Gastroenterology, Hospital Universitario Álvaro Cunqueiro, Vigo, Pontevedra, Spain; 38 Infectious Diseases Unit, Hospital Universitario Donostia, Donostia, Gipuzkoa, Spain; 39 Infectious Diseases Unit, Hospital Regional Universitario de Málaga, Málaga, Spain; 40 AbbVie Spain, S.L.U., Madrid, Spain; Middle East Liver Diseases (MELD) Center, ISLAMIC REPUBLIC OF IRAN

## Abstract

**Aim:**

We describe the effectiveness and safety of the interferon-free regimen ombitasvir/paritaprevir/ritonavir plus dasabuvir with or without ribavirin (OBV/PTV/r ± DSV ± RBV) in a nationwide representative sample of the hepatitis C virus (HCV) monoinfected and human immunodeficiency virus-1/hepatitis C virus (HIV/HCV) coinfected population in Spain.

**Material and methods:**

Data were collected from patients infected with HCV genotypes 1 or 4, with or without HIV-1 coinfection, treated with OBV/PTV/r ± DSV ± RBV at 61 Spanish sites within the initial implementation year of the first government-driven “National HCV plan.” Effectiveness was assessed by sustained virologic response at post-treatment week 12 (SVR12) and compared between monoinfected and coinfected patients using a non-inferiority margin of 5% and a 90% confidence interval (CI). Sociodemographic and clinical characteristics or patients and adverse events (AEs) were also recorded.

**Results:**

Overall, 2,408 patients were included in the intention-to-treat analysis: 386 (16%) were patients with HIV/HCV. Patient selection reflected the real distribution of patients treated in each participating region in Spain. From the total population, 96.6% (95% CI, 95.8–97.3%) achieved SVR12. Noninferiority of SVR12 in coinfected patients was met, with a difference between monoinfected and coinfected patients of −2.2% (90% CI, −4.5% - 0.2%). Only genotype 4 was associated with non-response to OBV/PTV/r ± DSV ± RBV treatment (*p*<0.001) in the multivariate analysis. Overall, 286 patients (11.9%) presented AEs potentially related to OBV/PTV/r ± DSV, whereas 347 (29.0%) presented AEs potentially related to ribavirin and 61 (5.1%) interrupted ribavirin.

**Conclusions:**

Our results confirm that OBV/PTV/r ± DSV ± RBV is effective and generally well tolerated in a representative sample of the HCV monoinfected and HCV/HIV coinfected population in Spain within the experience of a national strategic plan to tackle HCV.

## Introduction

In 2015, when this study was initiated, it was estimated that 71 million individuals were living with hepatitis C virus (HCV) infection worldwide, more than 2.2 million of whom were believed to be coinfected with human immunodeficiency virus (HIV)[1]. According to the World Health Organization, globally, approximately 71 million people have HCV infection [2]. In Europe, the prevalence of HCV ranges from 0.1% in Belgium, Ireland, and the Netherlands to 5.9% in Italy, while for Spain, the latest serologic survey estimates the overall prevalence of anti-HCV at approximately 1.1% of the Spanish population [3], lower than previously reported [4]. Notably, only 41% anti-HCV cases had positive HCV-RNA [3]. Most infections are caused by genotypes (GT) 1a, 1b, 2, 3 (almost exclusively 3a) and 4 [5]. Genotype 1b is the most prevalent (42.4%) in Spain, followed by 1a (22.5%), 3 (18.6%), 4 (10.6%) and 2 (4.6%) [6]. Coinfection with HIV is one of the most common comorbidities in patients with HCV [7]. In 2016, the prevalence of HCV in patients infected with HIV in Spain was 34.6% and, unlike HCV monoinfected patients, the most prevalent GTs in this population were GT1a and GT4 [8].

HCV infection can lead to many long-term complications. Most patients (80%–85%) who become acutely HCV-infected or HIV/HCV-coinfected progress to chronic HCV infection, and are at risk of developing cirrhosis, portal hypertension, hepatic decompensation and hepatocellular carcinoma[9]. Treating HCV infection prevents the progression of hepatic fibrosis to cirrhosis and the aforementioned complications, even with initial treatments based on peginterferon (peg-IFN) and ribavirin [9].

Interferon-free direct-acting antiviral agents (DAAs) have shown high effectiveness against HCV infection, with sustained virologic response (SVR) rates above 90% [10, 11]. Accordingly, they have become the new standard of care in patients with chronic HCV infection [10, 11]. The DAA combination of ombitasvir/paritaprevir/ritonavir plus dasabuvir with or without ribavirin (OBV/PTV/r ± DSV ± RBV) has shown favorable efficacy and tolerability in clinical studies [12–16].

In 2015, health authorities in Spain launched the first “National HCV Plan” to provide guidelines on the treatment of patients with already-diagnosed chronic HCV infection, which was designed to be implemented over the course of 3 years. According to this strategic plan, all patients with chronic HCV infection, whether monoinfected or coinfected with HIV, or whether they were treatment-naïve or had not responded to a prior antiviral treatment, were considered candidates for antiviral treatment. This treatment freely accessed by all patients insured under the Spanish National Health System.

Most HCV patients identified at that time were treated within the first year—between 2015 and 2016[17]. This plan was contemporaneous with the approval and market launch of OBV/PTV/r and DSV in Spain in April 2015 (Viekirax^®^, AbbVie Inc., North Chicago, IL, and Exviera^**®**^, AbbVie Deutschland GmbH & Co. KG, Ludwigshafen, Germany, respectively)[18, 19] to treat patients with chronic HCV GT1 and GT4 infections, regardless of HIV coinfection status [10, 11, 20]. Viekirax® [18] contains PTV/r and OBV. PTV is an inhibitor of HCV NS3/4A protease which is necessary for the proteolytic cleavage of the HCV encoded polyprotein and is essential for viral replication. OBV is administered with ritonavir (r), a potent CYP3A4 inhibitor used as a pharmacokinetic enhancer in order to achieve efficacious exposures. Exviera® [19], on the other hand, contains DSV, a non-nucleoside inhibitor of the HCV RNA-dependent RNA polymerase encoded by the NS5B gene.

This study included patients who had started treatment within the first year following the approval of Viekirax^®^ and Exviera^®^, and within the strategic plan for tackling HCV infection among residents in Spain universally treated by the Spanish National Health System. Preliminary real-world data with coexisting IFN-free DAAs have shown high rates of SVR in Spanish patients monoinfected with HCV GT1, as observed in registrational clinical trials[21, 22]. However, important subgroups of patients, such as those with HIV coinfection or GT4, were not included in these studies. The aim of this study was to describe the effectiveness and safety of the IFN-free OBV/PTV/r ± DSV ± RBV regimen in a large nationwide representative sample of the HCV monoinfected and HIV/HCV coinfected populations in Spain.

## Materials and methods

### Study design and patients

A non-interventional, retrospective, national, multicenter study was designed, with the participation of 61 sites in 48 hospitals distributed across all administrative regions of Spain (including 48 gastroenterology departments and 13 internal medicine/infectious diseases departments). The participating sites were public hospitals, located in any region of Spain, thus ensuring a heterogeneous patient sample and reducing the likelihood of a biased study population. Sites were selected taking into consideration the patient population under study and requirements to reach the weighted sample size per region. The information is listed in S1 Table, by regions, centers/sites/departments and specialties. S1 Table also includes the complete list of the centres which have contributed to the study.

Data were collected after a review of databases and medical records of adult patients with confirmed chronic HCV GT1 or GT4 infection, any stage of fibrosis, HCV monoinfection or HIV-1/HCV coinfection who had been treated with OBV/PTV/r ± DSV ± RBV. The decision to treat and the choice of treatment, including treatment duration and the use or not of concomitant RBV, had been at the discretion of the treating physician in the context of routine medical care. Patients included had initiated treatment from April 2015 to March 2016 and had ended it (complete treatment or premature termination) by June 2016. All HCV viral load determinations at week 12 after the end of treatment (or the last visit in case of rebound) had been performed by September 2016. Patients with hepatitis B coinfection, decompensated cirrhosis or who had participated in a concurrent interventional therapeutic trial while on treatment or 12 weeks after treatment with OBV/PTV/r ± DSV ± RBV were excluded.

The institutional review boards of all but three participating sites exempted the investigators from obtaining patient consent forms because the study was fully retrospective and had a short period of anonymous data collection. In the three sites where consent was required, informed consent was obtained in writing. No information identifying patients was captured. The study was approved by the Spanish Agency of Medicinal Products and Medical Devices (code ABB-OMD-2016-01) and obtained its first approval by the Ethic Committee of the Hospital la Princesa, Madrid with data 21 of July 2016. The study was presented to evaluation and/or notification to the rest of Ethic Committees of the hospitals involved, depending of the requirements of each center. The independent Ethic Committees that evaluated the study are listed in S1 Supporting information.

### Outcome-related variables

Sociodemographic variables, clinical characteristics of HCV infection, medical history, previous HCV therapy, HCV RNA count and laboratory tests, characteristics of the study treatment and adverse events (AEs) were collected. As this is an observational study, each treating physician followed standard clinical practice and verified that concomitant medication could be safely administered with the DAA regimen (including ritonavir) and RBV. Some medications were contraindicated and some required dose adjustments due to potential drug-drug interactions.

### Sustained virologic response at week 12 (SVR12)

Treatment effectiveness was measured by the percentage of patients achieving SVR12, defined as an HCV RNA below the lower limit of quantification or detection 12 weeks (70 to 126 days) after treatment completion. HCV RNA levels were determined using real-time polymerase chain reaction based on either the Roche COBAS TaqMan HCV or the Abbott RealTime HCV polymerase chain reaction assays. Patients were considered responders if HCV RNA was undetectable (lower limit of quantification or detection ≤50 IU/mL) or unquantifiable (lower limit of quantification ≤50 IU/mL). Virologic response was also assessed at the end of treatment (EoT).

To analyze the difference in SVR12 rates between patients with monoinfection and coinfection, both rates were compared assuming a non-inferiority margin of 5% (based on scientific discussion with researchers and clinicians) with a 90% confidence interval (CI) (Farrington-Manning method).

### Sample size estimation and control of inclusion bias

In the context of the strategic plan by the Spanish National Health System for tackling HCV [17], approximately 12 900 patients with HCV and HIV/HCV infections initiated treatment with PTV/r/OBV ± DSV ± RBV from April 2015 to March 2016. Expected effectiveness was estimated to be >90%, and a sample of 2,500 patients was considered sufficient to be representative of the population and to ensure an SVR12 rate precision of approximately 1.2%. To ensure a sample that is representative of the Spanish HCV scenario, the number of patients to be included from each administrative region was weighted to the proportion of patients who started treatment in that region during the defined period. After weighting, each participating site had an allotted number of patients to include, who were then randomly selected from their internal list of treated patients that fulfilled the inclusion criteria. If an institution had two participating departments (eg, hepatology, internal medicine), each unit had its own allotted number of patients to enroll and drew up an independent list of patients for random inclusion. Each confidential list was ordered chronologically per treatment start date, and an independent statistician provided each center or department with the randomly selected positions of the cases to include in the electronic Case Report Form.

### Statistical analysis

Qualitative variables were summarized in a table that included absolute and relative frequencies per group and in the whole population (column percentages, if no other specifications were detailed). Quantitative variables were provided using number of valid cases, mean, standard deviation, median, interquartile range, minimum, and maximum.

Quantitative parameters without a normal distribution and ordinal parameters were compared using a non-parametric Mann-Whitney test for two categories and Kruskal-Wallis test for three or more categories. Qualitative parameters were analyzed using a χ² test or, if assumptions were not met, Fisher’s exact test.

The significance level in bivariate analysis was generally established at a value of α = 0.05. Quantitative variables were compared between groups using a two-sample *t* test (when two groups were compared) and analysis of variance when three or more groups were compared. In cases where the analysis of variance test showed a significance level <0.05, the post hoc Bonferroni test was used at a significance level of 0.01 to detect differences in pairwise comparisons.

Univariate and multiple logistic regression was used to investigate the impact of the following explanatory covariates (patient and disease characteristics) at baseline on SVR12: HIV/HCV coinfection; demographic information (age, sex, ethnicity, body mass index, and IL28B polymorphism rs12979860); chronic HCV disease characteristics; HCV RNA level at baseline; HCV genotype/subtype; coinfections (HIV and HBV); comorbidities related or unrelated with the liver and chronic HCV; other comorbidities; alcohol use; clinical chemistry and hematology variables at baseline (alanine transaminase [ALT]/aspartate transaminase [AST] ratio, platelets, albumin and creatinine); in treatment-experienced patients (most recent previous treatment for chronic HCV and its outcome); OBV/PTV/r ± DSV ± RBV use (according or not to local label); and treatment adherence.

## Results

### Patient demographics and clinical characteristics at baseline

A total of 2,465 patients were registered from 61 sites across Spain, and 2,408 were included in the analysis as the core evaluable population fulfilling selection criteria: 2,022 patients with HCV and 386 patients HIV/HCV. Among coinfected patients, CD4 T-cell count was over 500 cells/mm3 in 63.2% (N = 244) at baseline and HIV-RNA was undetectable in 79.0% (N = 304). During follow-up, 65.3% (N = 252) of HIV coinfected patients achieved a CD4 count over 500 cells/mm3, while 81.8% of patients had undetectable HIV-RNA levels.

Patient selection reflected the real distribution of patients treated with OBV/PTV/r ± DSV ± RBV in each region in Spain. (S2 Table).

Table 1 shows the demographic and clinical characteristics of patients with chronic HCV included at baseline (n = 2,408); for the overall population, mean (SD) age was 57.8 (11.6) years and 59.8% were male. The mean duration of the disease was 15.7 (9.0) years. The most frequent genotype was GT1b (68.2%), followed by genotypes GT1a (19.0%) and GT4 (12.1%).

**Table 1:** Demographic and clinical characteristics of patients with Chronic HCV and HIV/HCV treated With OBV/PTV/r ± DSV ± RBV.

Variable	HCV	HIV/HCV	Total N = 2,408*
Department, n (%)			
Gastroenterology	1,981 (98.0)	25 (6.5)	2,006 (83.3)
Infectious diseases	41 (2.0)	361 (93.5)	402 (16.7)
Sex, male, n (%)	1,138 (56.3)	303 (78.5)^†^	1,441 (59.8)
Age, mean (SD), y	59.1 (11.9)	50.8 (6.4)^†^	57.8 (11.6)
<50 y, n (%)	415 (20.5)	129 (33.4)^†§^	544 (22.6)
≥50 y, n (%)	1,607 (79.5)	257 (66.6)	1,864 (77.4)
White ethnicity, n (%)	1,992 (98.5)	366 (95.6)^†§^	2,358 (98.0)
No alcohol consumption**, n (%)	1,525 (76.2)	278 (72.0)	1,803 (75.5)
BMI, mean (SD), Kg/m^2^	26.60 (4.1)	24.50 (3.8)^†^	26.1 (4.1)
BMI group, overweight, (BMI = 25−30 Kg/m^2^), n (%)	463 (45.3)	117 (35.7)	580 (43.0)
IL-28, genotype CC (SNP rs12979860), n (%)	201 (22.4)	83 (36.2)^†^	284 (25.2)
Duration of chronic HCV***, mean (SD), y	15.21 (9.1)	18.2 (8.2)^†^	15.7 (9.0)
HCV genotype, n (%)			
GT1	1,841 (91.0)	275 (71.2)^†§^	2,116 (87.9)
GT1a	284 (14.0)	173 (44.8)	457 (19.0)
GT1b	1,544 (76.4)	99 (25.6)	1,643 (68.2)
GT1 others	13 (0.6)	3 (0.8)	16 (0.7)
GT4	181 (9.0)	111 (28.8)	292 (12.1)
HCV RNA Quant-Test result, mean (SD), IU/mL	2 793 122.7 (4 301 803.4)	3 849 702.9 (5 999 880.7)	2 962 193.1 (4 630 442.9)
HCV RNA/Quant-Test, n (%)			
Abbott Real-Time HCV (RT-PCR)	443 (21.9)	53 (13.7)	496 (20.6)
Roche COBAS TaqMan HCV	1,252 (61.9)	203 (52.6)	1,455 (60.4)
HCV RNA Quant-Test result (global), n (%)			
Low HCV RNA (≤800 000 IU/mL)	670 (33.1)	110 (28.5)	780 (32.4)
High HCV RNA (>800 000 IU/mL)	1,352 (66.9)	276 (71.5)	1,628 (67.6)
Cirrhosis,**** n (%)	742 (36.7)	131 (33.9)	873 (36.3)
Cirrhosis (F4) per fibrosis grade method, n (%)			
Tested by Fibroscan^®^ (F4)	n = 1860 631 (33.9)	n = 373 123 (33.0)	n = 2,233 754 (33.8)
Biopsy (F4)	n = 81 30 (37.0)	n = 7 1 (14.3)	n = 88 31 (35.2)
Fibroscan^®^ (categorized),***** n (%)			
<8.8 kPa	613 (34.5)	130 (35.7)	743 (34.7)
8.8− < 9.6 kPa	146 (8.2)	27 (7.4)	173 (8.1)
9.6−< 12.5 kPa	386 (21.7)	74 (20.3)	460 (21.5)
12.5−< 14.6 kPa	153 (8.6)	31 (8.5)	184 (8.6)
14.6−< 20.0 kPa	181 (10.2)	44 (12.1)	225 (10.5)
>20.0 kPa+	298 (16.8)	58 (15.9)	356 (16.6)
Child Pugh Score, n (%)			
Class A (5−6)	713 (99.3)	119 (98.3)	832 (99.2)
Class B (7−9)	5 (0.7)	2 (1.7)	7 (0.8)
HIV infection, n (%)	—	386 (100)	386 (16)
Duration of HIV infection, mean (SD), y	—	21.9 (70)	21.9 (70)
ALT, mean (SD), IU/L	79.9 (60.5)	73.5 (61.4)^‡^	78.9 (60.6)
AST, mean (SD) IU/L	67.1 (46.5)	61.7 (40.6)	66.2 (45.7)
Total bilirubin, mean (SD), mg/dL	0.8 (0.5)	0.76 (0.5)	0.8 (0.5)
Hemoglobin, mean (SD) g/L	147.4 (16.5)	151.6 (15.8)	148.1 (16.5)
Platelets, mean (SD), ×10^9^/L	174.8 (67.9)	167.1 (63.6)	173.5 (67.2)
eGFR, mean (SD), mL/min/1.73m^2^***	89.5 (21.9)	93.2 (20.8)	90.1 (21.7)
Creatinine clearance, mean (SD), mL/min***	98.3 (34.9)	100.8 (30.9)	98.7 (34.3)
Most likely mode of HCV infection			
Drug use (i.v.)	192 (9.5)	305 (79.0)	497 (20.6)
Drug use (non i.v.)	18 (0.9)	2 (0.5)	20 (0.8)
Occupational*	25 (1.2)		25 (1.0)
Blood transfusion or transplantation	398 (19.7)	3 (0.8)	401 (16.7)
Perinatal	25 (1.2)	1 (0.3)	26 (1.1)
Contaminated medical device (other than i.v. drug use)	83 (4.1)		83 (3.4)
Heterosexual transmission	11 (0.5)	23 (6.0)	34 (1.4)
Homosexual transmission (MSM)	2 (0.1)	30 (7.8)	32 (1.3)
Other	29 (1.4)		29 (1.2)
Unknown	1239 (61.3)	22 (5.7)	1261 (52.4)
ART at baseline visit		357 (92.5)	357 (14.8)
Nucleosides and nucleotides excl. reverse transcriptase inhibitors		3 (0.8)	3 (0.1)
Protease inhibitors		144 (37.3)	144 (5.9)
Nucleoside and nucleotide reverse transcriptase inhibitors		98 (25.4)	98 (4.1)
Non-nucleoside reverse transcriptase inhibitors		52 (13.5)	52 (2.2)
Antivirals for treatment of HIV infections, combinations		223 (57.8)	223 (9.3)
Other antivirals		165 (42.7)	165 (6.9)

Abbreviations: ALT, alanine transaminase; APRI, aspartate transaminase to platelet ratio index; ART, Antiretroviral treatment; AST, aspartate transaminase; BMI, body mass index; eGFR, estimated glomerular filtration rate; GT, genotype; HCV, hepatitis C virus; HIV, human immunodeficiency virus; OBV/PTV/r ± DSV ± RBV, ombitasvir/paritaprevir/ritonavir plus dasabuvir with or without ribavirin; SNP, single nucleotide polymorphism.

^†^ *p*<0.0001;

^†§^ *p*<0.001 per distribution category;

^‡^ *p* = 0.0019.

* Not all patients had available data for all parameters.

**No alcohol consumption refers to the number of patients who reported that they were not consuming alcohol at that time of the study nor in the past.

***Duration of chronic HCV refers to the time from diagnosis of chronic HCV infection to the time of the study.

**** Cirrhosis level checked by the investigator based on invasive and/or non-invasive methods and clinical criteria. Regarding Fibroscan^®^ levels, most patients were considered as having fibrosis stage F4 (≥12.5 kPa) (n: 632 [35.6%] monoinfected, 133 [35.5%] coinfected and 765 [35.3%] of the total cases, respectively).

***** For renal function, creatinine clearance and eGFR were calculated by imputing mean weight in missing cases (77.3 Kg for men and 66.0 Kg for women).

The following differences were observed between patients with HIV/HCV and those with HCV: patients with HIV/HCV were predominantly males (78.5% vs. 56.3%) and younger (33.4% of HIV/HCV patients were <50 years vs. 20.5% of HCV patients, *p*<0.001 age distribution). Additionally, only 35.7% vs. 45.3% of patients with HIV/HCV vs. HCV, respectively, were overweight (body mass index >25–30 kg/m^2)^. Most patients in both groups were GT1 (HIV/HCV, 71.2%; HCV, 91.0%). However, the distribution of the sub-genotypes was different for each population, as follows: GT1b was more frequent in patients with HCV (76.4%), whereas GT1a was predominantly detected in patients with HIV/HCV (44.8%).

Most patients (92.7%) had the fibrosis grade determined by non-invasive methods (96% using transient elastography, Fibroscan^®^, EchoSens, Paris, France), and biopsy data were provided in 88 patients (3.7%). Cirrhosis was reported based on invasive/non-invasive methods and clinical guess in 873 patients (36.3%), and 7 cirrhotic patients (0.8%) had a Child-Pugh Score class B (7–9 points). See Table 1 for further details.

All patients (n = 2,408) had the HCV RNA quantification performed a mean of 17.7 weeks before the beginning of treatment. The mean HCV RNA at baseline was 2,962,193 IU/mL, and 67.6% of patients had HCV RNA higher than 800 000 IU/mL. A higher percentage of patients with HIV/HCV than HCV showed HCV RNA over 800 000 IU/mL (71.5 vs. 66.9, *p* = 0.074).

Mean (SD) laboratory values for patients with HCV (n = 1,970) and HIV/HCV (n = 384) were 98.3 (34.9) mL/min vs. 100.8 (30.9) mL/min for creatinine clearance and 89.5 (21.9) mL/min/1.73m^2^ vs. 93.2 (20.8) mL/min/1.73m^2^ for estimated glomerular filtration rate (eGFR), respectively.

Sixty-two percent of patients (n = 1,498) presented comorbidities (HCV: 62.9%; HIV/HCV: 58.5%), the most prevalent being cardiovascular diseases (30.4%), diabetes (14.2%) and psychiatric disorders (11.8%). Accordingly, 1,614 patients took concomitant medication (33% cardiovascular drugs, 29% alimentary drugs [mainly antacids] and 29% nervous system drugs). Patients with HIV/HCV received the following anchor antiretroviral therapies for the HIV infection: integrase inhibitors (41.2%), protease inhibitors (37.3%) or non-nucleoside reverse transcriptase inhibitors (13.74%).

### Previous treatment for HCV infection

A total of 1,025 patients (42.6%) were previously treated for HCV, the most frequent combinations being IFN or peg-IFN + RBV (81.0%). In addition, 42 patients (4.1%) had also received DAAs, as follows: 42.9% telaprevir, 21.4% boceprevir and 16.7% simeprevir. More than 40% of previously treated patients (HCV: 43.6%; HIV/HCV: 40.9%) were null responders, 22.1% had relapsed (HCV: 21.8% and HIV/HCV: 23.9%), 12.6% had partial response (HCV: HCV: 12.9% and HIV/HCV: 10.7%) and finally, 2.1% had breakthrough (HCV: 1.7% and HIV/HCV: 4.4%).

### Treatment regimen and duration

Overall, most patients (79.5%) received 12 weeks of OBV/PTV/r + DSV treatment, with (31%) or without (48.5%) concomitant ribavirin, followed by an OBV/PTV/r regimen for 12 weeks (9.1%) and adding ribavirin (8.1%). The remaining patients received treatment for 24 weeks (11.3%). The treatment regimen was adjusted according to the patient’s genotype and cirrhosis status (Table 2): OBV/PTV/r + DSV 12 weeks was the most frequent in GT1b without cirrhosis (91%), whereas most patients with GT1b with compensated cirrhosis had OBV/PTV/r + DSV plus ribavirin for 12 weeks (64%). Moreover, most patients with GT4 without cirrhosis received OBV/PTV/r + RBV for 12 weeks (84.8%). For patients with GT4 and compensated cirrhosis, the most common treatment duration was 24 weeks. The planned study regimen for most patients with HCV was OBV/PTV/r + DSV ± RBV for 12 weeks (84.6%) (only 36.3% added ribavirin). For patients with HIV/HCV, 53.1% were eligible for OBV/PTV/r + DSV ± RBV for 12 weeks and the addition of ribavirin (61.0%). Overall, a total of 218 patients with HCV (9.1%) were treated off-label, that is, with OBV/PTV/r ± DSV ± RBV regimens different from those included on the label or national guidelines (Table 2). Notably, those off-label patients included 20.3% of the 74 patients with HCV GT4 and compensated cirrhosis.

For treatments with a planned duration of 12 weeks, the real duration was within 1 week of that planned in 96.2% of patients. In the case of 24 weeks, 89.0% of patients with HCV had a similar duration (±1 week). The main reasons for deviating treatment durations were AEs (23.4%) and other unspecified reasons.

**Table 2:** Selected treatment regimens.

Treatment Regimen		GT1b n = 1,654, n (%)		GT1a n = 455, n (%)		GT4 n = 291, n (%)		Other, n (%) n = 11	Total, n (%) N = 2,408
		Without Cirrhosis n = 1,037	With Compensated Cirrhosis* n = 614	Without Cirrhosis n = 278	With Compensated Cirrhosis n = 177	Without Cirrhosis n = 217	With Compensated Cirrhosis n = 74		
Regimen received	OBV/PTV/r + RBV 12 weeks		1 (0.2)		1 (0.6)	**184 (84.8)**	10 (13.5)		196 (8.1)
	OBV/PTV/r 12 weeks	18 (1.7)	2 (0.3)			3 (1.4)	1 (1.4)		24 (1.0)
	OBV/PTV/r + RBV 24 weeks		1 (0.2)		1 (0.6)	11 (5.1)	**59 (79.7)**	1 (9.1)	73 (3.0)
	OBV/PTV/r + DSV + RBV 12 weeks	66 (6.4)	**393 (64.0)**	**244 (87.8)**	23 (13.0)	13 (6.0)		7 (63.6)	746 (31.0)
	OBV/PTV/r + DSV 12 weeks	**944 (91.0)**	**205 (33.4)**	15 (5.4)	1 (0.6)	4 (1.8)			1,169 (48.5)
	OBV/PTV/r + DSV + RBV 24 weeks	3 (0.3)	9 (1.5)	13 (4.7)	**150 (84.7)**	2 (0.9)	3 (4.1)	3 (27.3)	183 (7.6)
	OBV/PTV/r + DSV 24 weeks	6 (0.6)	3 (0.5)	6 (2.2)	1 (0.6)		1 (1.4)		17 (0.7)
On-label treatment**	Yes	**944 (91.0)**	**598 (97.4)**	**244 (87.8)**	**150 (84.7)**	**184 (84.8)**	**59 (79.7)**	11 (100)	2,190 (90.9)
	No	93 (9.0)	16 (2.6)	34 (12.2)	27 (15.3)	33 (15.2)	15 (20.3)		218 (9.1)

Abbreviations: DSV, dasabuvir; GT, genotype; OBV, ombitasvir; PTV, paritaprevir; r, ritonavir; RBV, ribavirin.

* Note: Although OBV/PTV/r ± DSV has no indication in patients with decompensated cirrhosis, some patients with decompensated cirrhosis have been included according to the Child-Pugh Score. Use of OBV/PTV/r + DSV regarding treatment duration and concomitant use of RBV based on approved label is set in bold; summary per groups is shown here for on- and off-label treatment.

** The term on-label refers to the treatment that has been administer according to the EU Summary of Product Characteristics (SmPC).

### Virologic response

**Fig 1:**
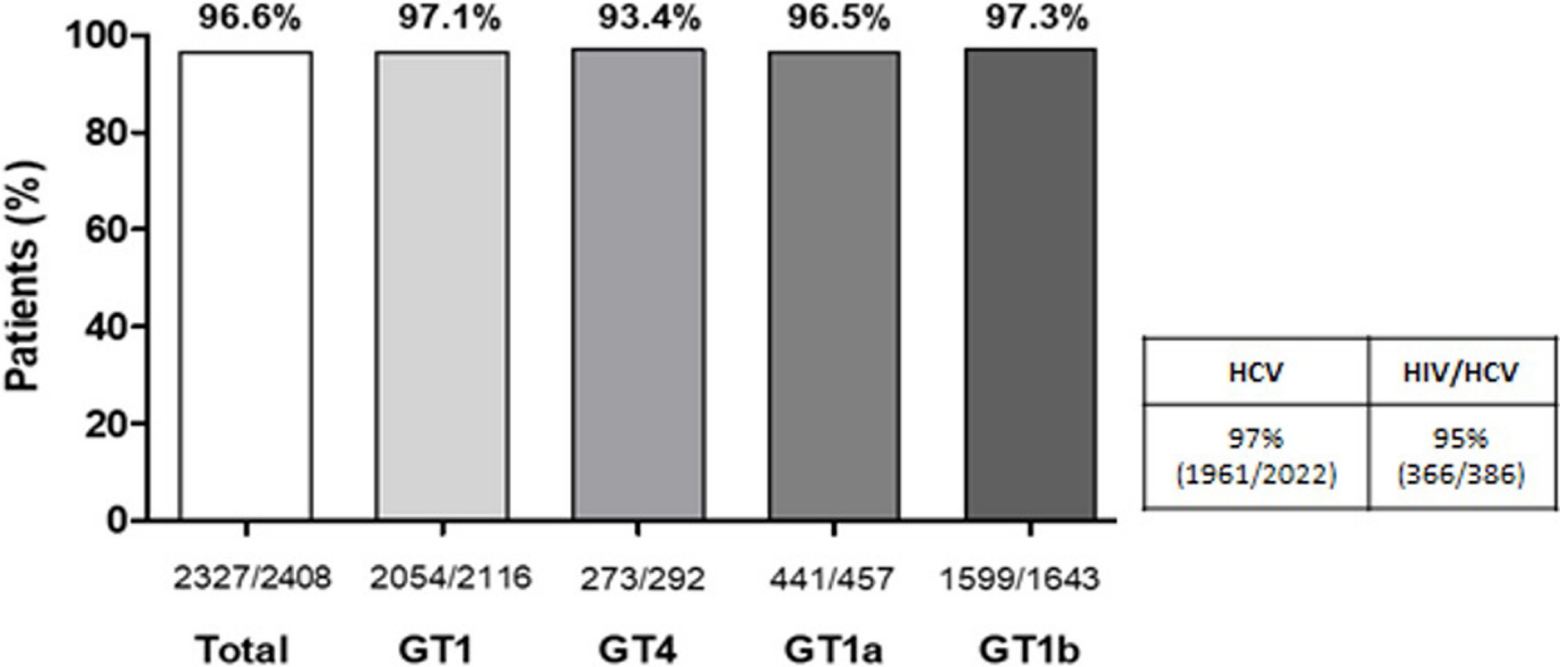
SVR12 according to genotype (ITT analysis). Abbreviations: GT, genotype; SVR12, sustained virologic response at 12 weeks; ITT, intention-to-treat. Abbreviations: GT, genotype; HCV, hepatitis C virus; HIV, human immunodeficiency virus.

At EoT, a total of 2,333 patients (96.9%) had virologic response, and only 17 had a detectable viral load. Among the intention-to-treat (ITT) population, 2,327 patients achieved SVR12 (96.6% [95% CI, 95.8−97.3%]). SVR12 was reached by 2,054 of 2,116 patients (97.1%) with GT1 (GT1a/G1x: 96.2%, GT1b: 97.3%) and 273 of 292 patients (93.5%) with HCV GT4 infection (Fig 1). When examining per cohort, SVR12 rates were 94.8% (95% CI, 92.1−96.8%) for patients with HIV/HCV and 97.0% (95%CI: 96.1−97.7%) for patients with HCV. Of the 81 patients (3.4%) who did not achieve SVR12, 44 (1.8%) had virologic failure: 17 relapses, 5 breakthroughs and 22 patients were finally confirmed as null responders (HCV RNA value ≥ 50 IU/mL). Failure to achieve SVR12 was not associated with virologic reasons (relapses, breakthroughs or failure to suppress) in 37 patients (45.6%) (S3 Table).

The non-inferiority analysis showed that patients with HIV/HCV had an SVR12 rate within the non-inferiority margin of 5% from the rate of patients with HCV (−2.2% [90% CI, −4.5% to 0.2%]).

Table 3 shows SVR12 rates and 95% CIs according to each ITT criterion in patients with HCV and those with HIV/HCV. Differences in rates between groups ranged from 1.8% to 2.6%.

**Table 3:** SVR12 According to HIV coinfection status (ITT analysis).

	HCV (83.97%)	HIV/HCV (16.03%)	Total (100%)	Adjusted Total*** SVR12	Noni-Inferiority 90%IC
ITT	1,961/2,022 (97.0%) [96.1–97.7%]	366/386 (94.8%) [92.1–96.8%]	2,327/2,408 (96.6%) [95.8–97.3%]	96.6% [95.9–97.3%]	-2.2% [-4.5% to 0.2%]
mITT1*	1,961/2,009 (97.6%) [96.8–98.2%]	366/385 (95.1%) [92.4–97.0%]	2,327/2,394 (97.2%) [96.5–97.8%]	97.2% [96.5–97.8%]	-2.6% [-4.9% to -0.3%]
mITT2**	1,961/1,994 (98.3%) [97.7–98.9%]	366/379 (96.6%) [94.2–98.2%]	2,327/2,373 (98.1%) [97.4–98.6%]	98.0% [97.5–98.6%]	-1.8% [-3.7% to 0.1%]

Abbreviations: HCV, hepatitis C virus; HIV, human immunodeficiency virus; mITT modified intention-to-treat; SVR12, sustained virologic response at 12 weeks.

* ITT modified criteria (mITT1) excludes patients with no response due to loss of follow-up.

** ITT modified criteria (mITT2) excludes patients with no response due to loss of follow-up and patients with other non-virologic reasons for missing data 70 days post-treatment.

*** The SVR12 analysis was adjusted according to the estimated proportion of HIV/HCV (82% and 18% of HCV and HIV/HCV patients, respectively).

### Multivariable logistic model for SVR12

According to the univariate analysis and considering the ITT population, the following baseline characteristics were significantly associated with lower SVR12 rates: age (<60 years; *p* = 0.01), HIV/HCV coinfection (*p* = 0.03), genotype 4 (*p* = 0.001) and prothrombin time >14 seconds (*p* = 0.008). Nevertheless, the multivariate analysis revealed that only the viral genotype was associated with non-response to treatment. GT4 was predictive of non-response to OBV/PTV/r + DSV ± RBV treatment (odds ratio, 2.6; 95% CI, 1.5–4.6; *p*<0.001).

### Safety

The AEs most frequently reported in the total population (n = 2,408) were anemia (18.6%), asthenia (13.2%), headache (6.0%), pruritus (5.9%), insomnia (5.2%), nausea (2.9%), diarrhea (2.8%) and fatigue (2.3%). Treatment-emergent AEs occurred in 24.8% of patients and only 2.0% presented serious AEs.

Overall, 286 patients (11.9%) presented AEs potentially related to OBV/PTV/r and PTV/r/OBV + DSV ± RBV, whereas 347 patients (14.4%) presented AEs potentially-related only to RBV. As a consequence, 29 patients (1.2%) had to discontinue OBV/PTV/r ± DSV treatment and 61 (2.5%) had to discontinue RBV. In addition, a decrease in dosage or temporary treatment interruption was proposed for 177 patients (7.4%) because of AEs.

Only 48 patients (2%) presented a serious AE, as follows: 56 serious AEs, including five hepatocellular carcinoma (0.2%), and eight deaths (0.3%), two considered possibly related to the study drug (pneumonia lasting 50 weeks and worsening of chronic renal disease) (Table 4).

**Table 4:** AEs Occurring during treatment or follow-up in patients treated With OBV/PTV/r ± DSV ± RBV.

AE	PTV/r/OBV + RBV n = 269	PTV/r/OBV + DSV + RBV n = 929	PTV/r/OBV + DSV n = 1,186	Total N = 2,408
Any AE	77 (28.6)	338 (36.4)	180 (15.2)	596 (24.8)
Severity	3 (1.1)	19 (2.0)	17 (1.4)	39 (1.6)
Any serious AE*	1 (0.4)	22 (2.4)	25 (2.1)	48 (2.0)
Death	0	3 (0.3)	5 (0.4)	8 (0.3)
AEs related to OBV/PTV/r ± DSV**	40 (14.9)	127 (13.7)	118 (9.9)	286 (11.9)
OBV/PTV/r ± DSV discontinued	2 (0.7)	17 (1.8)	10 (0.8)	29 (4.9)
AEs related to RBV*	64 (23.8)	283 (30.5)	N/A	347 (14.4)
RBV discontinued	4 (1.5)	57 (6.1)	N/A	61 (5.1)**
RBV reduced/interrupted	31 (11.5)	146 (15.7)	N/A	177 (14.8)**
AEs related to RBV	64 (23.8)	283 (30.5)	N/A	347 (29.0)**

Abbreviations: AE, adverse event; OBV/PTV/r ± DSV ± RBV, ombitasvir/paritaprevir/ritonavir plus dasabuvir with or without ribavirin.

Values are presented as n (%).

* There were 56 serious AEs, 5 probably/possibly related to OBV/PTV/r + DSV + RBV, 10 with OBV/PTV/r + DSV and 7 with RBV.

** Calculated over total of patients treated with RBV.

The most common AEs related to OBV/PTV/r ± DSV were asthenia (19.4%), headache (10.0%), insomnia (10.0%) and pruritus (8.6%), while AEs related to RBV were anemia (37.5%), asthenia (14.4%), pruritus (7.4%), insomnia (4.6%) and rash (3.8%).

Levels of ALT showed a statistically significant decrease at EoT (mean [SD] decrease of 58.4 [61.4] IU/mL) and 12 weeks after EoT (mean [SD] decrease of 57.8 [58.1] IU/mL) compared with baseline levels (*p*<0.001). Additionally, a significant decrease (*p*<0.001) in AST activity was observed from baseline levels to EoT (mean [SD] decrease of 43.3 [44.1] IU/mL) and 12 weeks after EoT (mean [SD] decrease of 42.3 [42.9] IU/mL). AST/ALT ratio levels showed a statistically significant decrease at EoT (mean [SD] decrease of 0.8 [0.9] IU/mL) and 12 weeks after EoT (mean [SD] decrease of 0.8 [0.8] IU/mL) compared with the baseline value.

Conversely, although mean hemoglobin values decreased during the treatment period (mean of 14.8 g/dL at baseline and 13.7g/dL at EoT), they recovered 12 weeks after EoT (14.7 g/dL). Mean eGFR decreased slightly throughout treatment (90.5 [31.0] mL/min/1.73m^2^ at baseline and 89.1 [21.6] mL/min/1.73m^2^ at EoT) and 12 weeks after EoT (88.2 [21.3] mL/min/1.73m^2^).

## Discussion

To the best of our knowledge, this study is one of the largest describing the effectiveness of the OBV/PTV/r ± DSV ± RBV regimen under routine clinical conditions in both patients with HCV monoinfection and patients with HIV/HCV coinfection. Both cohorts included patients with HCV GT1 and GT4 infection, cirrhosis and patients who had received previous HCV treatment. Of note, this study was conducted in the context of a government-driven HCV strategic plan aimed at reducing the morbidity and mortality caused by HCV in Spain[17], conceived in view of the launch of new DAAs against hepatitis C on the market, including OBV/PTV/r ± DSV ± RBV[18, 19]. As a result of this national plan, a remarkably large number of patients with HCV and HIV/HCV started OBV/PTV/r ± DSV ± RBV treatment in 2015, its first year of implementation. To design a study that adequately reflected the significance and extent of this approach against hepatitis C, we considered it paramount to apply a distinct method to recruit a representative sample of patients at a national level. A relevant randomized sample of patients who were just initiating treatment with OBV/PTV/r ± DSV ± RBV from April 2015 to March 2016 was therefore selected in each health administration area in Spain. Thus, we believe our results accurately reflect real clinical practice at this crucial moment and at a national level.

In the study population, treatment was generally based on the label recommendations for patients with infection by HCV genotypes 1a, 1b or 4, regardless of HIV-1 status [10, 11, 20]. Only 9.1% of patients with HCV were not treated according to local guidelines. This group did not include, for instance, patients with GT1b and compensated cirrhosis, treated for 12 weeks with OBV/PTV/r + DSV but without ribavirin—at that time not yet on the label—based on already published data from the TURQUOISE III study [14]. Thus, OBV/PTV/r ± DSV based regimens included on the label after study patients had been treated, but with published clinical trial data, have not been considered off-label. Nevertheless, there were no patients GT1b-naive treated for 8 weeks with OBV/PTV/r ± DSV.

Therapy with DAA with OBV/PTV/r ± DSV ± RBV has been extensively studied in clinical trials and observational studies and has shown high effectiveness, with overall SVR12 rates ranging from 95% to 100%. Our findings in real-world clinical practice were comparable with those in clinical trials [14, 22–26] and previous post-marketing studies [27, 28] that reported SVR12 rates of ≥95% across different patient subgroups. A recent real-life study has even shown the durability of the SVR response after 2 years following the EoT with OBV/PTV/r ± DSV ± RBV [29].

The results of this study also showed that, although patients with HIV/HCV coinfection (94.8% [95% CI: 92.1–96.8%]) had lower SVR12 rates than patients with HCV monoinfection (97.0% [95% CI: 96.1–97.7%]), clinical effectiveness in the coinfected group, as measured by SVR12, was statistically non-inferior to that of the monoinfected group. Our SVR12 rates are comparable with those reported for patients with HCV monoinfection (94% [95% CI: 91.7–96%]) and those with HIV/HCV coinfection (97% [95%CI: 95.7–99.4%]) with all-oral DAA regimens in a recent real-life study conducted in a similar context in Spain [30].

Of note, from the 386 patients with HIV/HCV included in this study, only 20 did not achieve SVR12 (5.2%, 6 with missing data). Patients with HIV/HCV with or without cirrhosis treated with other DAAs have achieved high SVR12 rates, which shows that DAA regimens are effective and well tolerated by patients coinfected with HIV/HCV with or without cirrhosis, as previously reported [16, 31].

Among our patients with GT1, SVR12 rates were similar to those observed in a real-world study in a German cohort [28] and in a meta-analysis of the effectiveness of OBV/PTV/r ± DSV ± RBV [27], which reported overall SVR12 rates above 95% for patients with GT1. However, the SVR12 rates in our study for GT4 patients were somewhat lower than those reported in those studies (>95%) [27, 28]. Moreover, in our study, only GT4 was predictive of OBV/PTV/r ± RBV treatment failure, possibly because a higher percentage of GT4 patients with compensated cirrhosis (20.3%) were treated off-label (OBV/PTV/r ± RBV 12 weeks; OBV/PTV/r + DSV ± RBV 24 weeks) in comparison with patients with GT1b (2.6%) and GT1a (15.3%). In addition, our GT4 group included a high percentage of patients with HIV/HCV (38.0%) in whom SVR rates were generally lower than in patients with HCV. However, in our study, coinfection with HIV was not an independent predictor of treatment failure.

Additionally, failure to achieve SVR12 in our population was mostly associated with reasons not related to viral infection control, such as missing data or loss of follow-up, but not with virologic failure. The combination of OBV/PTV/r ± DSV ± RBV was generally well tolerated. In fact, only one in four patients (24.8%) reported an AE, slightly lower than those seen in clinical studies (52% in the German study [28] and 77%–78% in clinical trials [32]), but similar to the rates reported in a recent global study [33]. Nevertheless, the observational and retrospective nature of this study may have led to potential underreporting of AEs.

In our study, only 29 patients (4.9%) interrupted the OBV/PTV/r ± DSV regimen, while 61 patients (5.1%) treated with ribavirin abandoned prematurely. The most common treatment-related AEs reported are similar to results obtained in clinical trials [14], with anemia being the most frequent AE reported in the ribavirin-treated group. In addition, a slight decline in the eGFR was recorded from baseline to 12 weeks post-treatment, but the mean reduction in the eGFR was <10 mL/min/1.73m^2^, which is considered the minimum change that could be associated with meaningful clinical significance.

From a methodological perspective, our study provides added value to previous observational studies, derived from the sample size and the particular method for patient selection to obtain a broad and representative HCV and HIV/HCV population, randomly selected from a large number of study sites in Spain. However, because of the real-world design of the study, some general limitations of observational cohorts might also apply here. For example, the treatment regimen and duration were determined according to the criteria of the physician, and the 9.1% of patients treated off-label were not independently analyzed, nor was this variable included in the regression model analysis. Monitoring of patients during follow-up (including laboratory assessment) was also conducted at the investigator’s discretion, resulting in missing data for some patients. In addition, local practice discrepancies or data entry errors might have occurred.

These results support and complement previous clinical and real-world data confirming that OBV/PTV/r ± DSV ± RBV is effective and generally well tolerated in patients with chronic HCV monoinfection and HIV/HCV coinfection under routine practice conditions. In addition, our study provides an interesting insight into the outcomes of DAAs in Spain within the experience of a well-articulated national strategic plan to tackle HCV, one of the main public health problems in Spain.

## Supporting information

S1 Supporting information List of institutional ethic committees. (DOCX)

S1 Table List of centers included in the study. Abbreviations: Gastro, gastroenterology department; Infecc, Internal medicine/infectious diseases department.
(DOCX)

S2 Table Distribution of patients treated with OBV/PTV/r ± DSV ± RBV in each region. Abbreviations: HCV, hepatitis C virus; HIV, human immunodeficiency virus; OBV/PTV/r ± DSV ± RBV, ombitasvir/paritaprevir/ritonavir plus dasabuvir with or without ribavirin. Sample values are presented as n (%); Real Distrib. values are presented at %.
(DOCX)

S3 Table Rates of virologic response and virologic failure to OBV/PTV/r ± DSV ± RBV (ITT analysis). Abbreviations: HCV, hepatitis C virus; HIV, human immunodeficiency virus; ITT, intention-to-treat; OBV/PTV/r ± DSV ± RBV, ombitasvir/paritaprevir/ritonavir plus dasabuvir with or without ribavirin; SVR12, sustained virologic response at 12 weeks; VR EoT, virologic response at end of treatment. Values are presented as n (%).
(DOCX)
