# Supplementary material for: Real-world evidence of the effectiveness of ombitasvir-paritaprevir/r ± dasabuvir ± ribavirin in patients monoinfected with chronic hepatitis C or coinfected with human immunodeficiency virus-1 in Spain
Source: PLoS One. 2019 Nov 12;14(11):e0225061. doi: 10.1371/journal.pone.0225061 (PMC6850697; doi:10.1371/journal.pone.0225061)
Supplement: S1 Supporting information — (DOCX) [file pone.0225061.s001.docx]

**S1 Supporting information**. List of Institutional ethic committees

- Comité de Ética de la investigación biomédica provincial de Granada, Granada, Andalusia, Spain.
- Comité de Ética de la Investigación del Principado de Asturias, Asturias, Spain.
- Comité Ético de Investigación Clínica de la Comunidad Autónoma del País Vasco (CEIC-E), Basque Country, Spain.
- Comité de Ética de la Investigación del Hospital Universitario de Canarias, Santa Cruz de Tenerife, Canary Islands, Spain.
- Comité de Ética de la Investigación del Hospital Universitario Germans Trias i Pujol, Barcelona, Catalonia, Spain.
- Comité Ético de Investigación Clínica del Hospital Clínic de Barcelona, Barcelona, Catalonia, Spain.
- Comité Ético de Investigación Clínica de la Corporació Sanitària Parc Taulí de Sabadell, Barcelona, Catalonia, Spain.
- Comité Ético de Investigación Clínica de la Fundació de Gestió Sanitària del Hospital de la Santa Creu i Sant Pau de Barcelona, Barcelona, Catalonia, Spain.
- Comité Ético de Investigación Clínica Parc de Salut Mar, Barcelona, Catalonia, Spain.
- Comité de Ética de la Investigación Clínica CEI Girona, Girona, Catalonia, Spain.
- Comité Ético de Investigación Clínica del Hospital Universitari de Tarragona Joan XXIII, Tarragona, Catalonia, Spain.
- Comité de Ética de la Investigación clínica del Complejo hospitalario de Albacete y su área de salud, Albacete, Castile-La Mancha, Spain.
- Comité Ético de Investigación Clínica de la gerencia de Atención Integrada de Ciudad Real, Ciudad Real, Castile-La Mancha, Spain.
- Comité Ético de Investigación Clínica del Complejo Hospitalario de Toledo, Toledo, Castile-La Mancha, Spain.
- Comité Ético de Investigación Clínica de León, León, Castile-Leon, Spain.
- Comité Ético de Investigación Clínica del Área de Salud Valladolid Oeste, Valladolid, Castile-Leon, Spain.
- Comité de Ética de la Investigación de la Fundación Jiménez Díaz, Madrid, Madrid Community, Spain.
- Comité Ético de Investigación Clínica Hospital General Universitario Gregorio Marañón, Madrid, Spain.
- Comité Ético de Investigación Clínica del Hospital Universitario de la Princesa, Madrid, Madrid Community, Spain.
- Comité Ético de Investigación Clínica del Hospital Clínico San Carlos de Madrid, Madrid, Madrid Community, Spain.
- Comité ético de Investigación Clínica del Hospital Universitario La Paz, Madrid, Madrid Community, Spain.
- Comité Ético de Investigación con medicamentos del Hospital Universitario Puerta de Hierro Majadahonda de Madrid, Madrid, Madrid Community, Spain.
- Comité Ético de Investigación Clínica de Navarra, Navarra, Navarra, Spain.
- Comité Ético de Investigación CEI Hospital Arnau de Vilanova de Valencia, Valencia, Valencian Community, Spain.
- Comité Ético de Investigación Clínica del Hospital Universitario y Politécnico La Fe, Valencia, Valencian Community, Spain.
