# Supplementary material for: Real-world evidence of the effectiveness of ombitasvir-paritaprevir/r ± dasabuvir ± ribavirin in patients monoinfected with chronic hepatitis C or coinfected with human immunodeficiency virus-1 in Spain
Source: PLoS One. 2019 Nov 12;14(11):e0225061. doi: 10.1371/journal.pone.0225061 (PMC6850697; doi:10.1371/journal.pone.0225061)
Supplement: S1 Table — Abbreviations: Gastro, gastroenterology department; Infecc, Internal medicine/infectious diseases department. (DOCX) [file pone.0225061.s002.docx]

**S1 Table. List of centers included in the study**

| **Region** | **#** | **Center** | **Gastro** | **Infecc** |
| --- | --- | --- | --- | --- |
| AND | 1 | Complejo Hospitalario Universitario de Granada | X |  |
|  | 2 | H. Puerta del Mar | X |  |
|  | 3 | H. Regional Universitario de Málaga | X | X |
|  | 4 | H. Universitario Reina Sofía | X |  |
|  | 5 | H. Universitario Virgen del Rocío | X | X |
|  | 6 | H. Universitario Virgen de la Victoria | X |  |
| ARA | 7 | H. Clínico Universitario Lozano Blesa | X |  |
| AST | 8 | H. Universitario Central de Asturias | X |  |
| BAL | 9 | H. Universitario Son Espases | X |  |
| CANAR | 10 | H. Universitario de Canarias | X |  |
| CANT | 11 | H. Universitario Marqués de Valdecilla | X |  |
| CAT | 12 | H. Clínic de Barcelona | X | X |
|  | 13 | H. Del Mar | X |  |
|  | 14 | H. De la Santa Creu i Sant Pau | X |  |
|  | 15 | H. Universitari de Girona Doctor Josep Trueta | X |  |
|  | 16 | H. Universitari Germans Trias i Pujol | X | X |
|  | 17 | H. Universitari Joan XXIII | X |  |
|  | 18 | H. Universitari Parc Taulí | X |  |
| CLM | 19 | Complejo Hospitalario Universitario de Albacete | X |  |
|  | 20 | Hospital General Universitario de Ciudad Real | X |  |
|  | 21 | Hospital Virgen de la Salud | X |  |
| CyL | 22 | H. De León | X |  |
|  | 23 | H. Universitario de Burgos | X |  |
|  | 24 | H. Universitario Río Hortega | X |  |
| GAL | 25 | Complejo Hospitalario Universitario A Coruña | X |  |
|  | 26 | Complejo Hospitalario Universitario de Pontevedra | X |  |
|  | 27 | H. Universitario Álvaro Cunqueiro | X | X |
| MAD | 28 | Fundación Hospital Alcorcón | X |  |
|  | 29 | H. 12 de Octubre | X | X |
|  | 30 | H. Clínico San Carlos | X |  |
|  | 31 | H. General Universitario Gregorio Marañón | X | X |
|  | 32 | H. Universitario de la Princesa | X |  |
|  | 33 | H. Universitario Fundación Jiménez Díaz | X |  |
|  | 34 | H. Universitario La Paz | X | X |
|  | 35 | H. Universitario Príncipe de Asturias | X |  |
|  | 36 | H. Universitario Puerta de Hierro | X |  |
|  | 37 | H. Universitario Ramón y Cajal | X | X |
| MUR | 38 | H. Universitario Santa Lucía | X |  |
| NAV | 39 | Complejo Hospitalario de Navarra | X |  |
| PV | 40 | H. De Basurto | X | X |
|  | 41 | H. De Cruces | X |  |
|  | 42 | H. Universitario de Araba – Txagorritxu | X | X |
|  | 43 | H. Universitario de Galdakao | X |  |
|  | 44 | H. Universitario Donostia | X | X |
| VAL | 45 | H. Arnau de Vilanova | X |  |
|  | 46 | H. General Universitario de Alicante | X | X |
|  | 47 | H. Universitario Doctor Peset | X |  |
|  | 48 | H. Universitario y Politécnico La Fe | X |  |

Abbreviations: Gastro, gastroenterology department; Infecc, Internal medicine/infectious diseases department
