# Supplementary material for: Real-world evidence of the effectiveness of ombitasvir-paritaprevir/r ± dasabuvir ± ribavirin in patients monoinfected with chronic hepatitis C or coinfected with human immunodeficiency virus-1 in Spain
Source: PLoS One. 2019 Nov 12;14(11):e0225061. doi: 10.1371/journal.pone.0225061 (PMC6850697; doi:10.1371/journal.pone.0225061)
Supplement: S2 Table — Abbreviations: HCV, hepatitis C virus; HIV, human immunodeficiency virus; OBV/PTV/r ± DSV ± RBV, ombitasvir/paritaprevir/ritonavir plus dasabuvir with or without ribavirin. Sample values are presented as n (%); Real Distrib. values are presented at %. (DOCX) [file pone.0225061.s003.docx]

**S2 Table**. **Distribution of patients treated with the PTV/r/OMV±DSV ± RBV in each region**

|  | **HCV** | | **HIV/HCV** | | **Total** | |
| --- | --- | --- | --- | --- | --- | --- |
| **Region** | **Sample** | **Real**  **Distrib.** | **Sample** | **Real**  **Distrib.** | **Sample** | **Real**  **Distrib.** |
| **Total** | **2022 (100%)** | **100%** | **386 (100%)** | **100%** | **2408 (100%)** | **100%** |
| Andalusia (+ Extremadura) | 338 (16.7%) | 15.9% | 71 (18.4%) | 19.3% | 409 (17.0%) | 15.9% |
| Aragon | 23 (1.1%) | 1.2% | 1 (0.3%) |  | 24 (1.0%) | 1.2% |
| Asturias | 27 (1.3%) | 1.3% |  |  | 27 (1.1%) | 1.3% |
| Balearic Islands | 45 (2.2%) | 2.2% | 12 (3.1%) | 2.7% | 57 (2.4%) | 2.2% |
| Canary Islands | 50 (2.5%) | 2.4% |  |  | 50 (2.1%) | 2.4% |
| Cantabria | 32 (1.6%) | 1.6% |  |  | 32 (1.3%) | 1.6% |
| Catalonia | 290 (14.3%) | 16.5% | 80 (20.7%) | 20.0% | 370 (15.4%) | 16.5% |
| Castile-La Mancha | 77 (3.8%) | 4.1% | 1 (0.3%) |  | 78 (3.2%) | 4.1% |
| Castile-Leon | 85 (4.2%) | 4.1% |  |  | 85 (3.5%) | 4.1% |
| Galicia | 113 (5.6%) | 5.6% | 5 (1.3%) | 6.7% | 118 (4.9%) | 5.6% |
| Madrid Community | 572 (28.3%) | 27.5% | 163 (42.2%) | 33.3% | 735 (30.5%) | 27.5% |
| Murcia Region | 27 (1.3%) | 1.3% |  |  | 27 (1.1%) | 1.3% |
| Navarre | 30 (1.5%) | 1.5% |  |  | 30 (1.2%) | 1.5% |
| Basque country (+ Rioja) | 137 (6.8%) | 6.7% | 41 (10.6%) | 8.1% | 178 (7.4%) | 6.6% |
| Valencian Community | 176 (8.7%) | 8.2% | 12 (3.1%) | 9.9% | 188 (7.8%) | 8.2% |

*Abbreviations: HCV (Hepatitis C virus); HIV (Human Immunodeficiency Virus)*
