# Supplementary material for: Real-world evidence of the effectiveness of ombitasvir-paritaprevir/r ± dasabuvir ± ribavirin in patients monoinfected with chronic hepatitis C or coinfected with human immunodeficiency virus-1 in Spain
Source: PLoS One. 2019 Nov 12;14(11):e0225061. doi: 10.1371/journal.pone.0225061 (PMC6850697; doi:10.1371/journal.pone.0225061)
Supplement: S3 Table — Abbreviations: HCV, hepatitis C virus; HIV, human immunodeficiency virus; ITT, intention-to-treat; OBV/PTV/r ± DSV ± RBV, ombitasvir/paritaprevir/ritonavir plus dasabuvir with or without ribavirin; SVR12, sustained virologic response at 12 weeks; VR EoT, virologic response at end of treatment. Values are presented as n (%). (DOCX) [file pone.0225061.s004.docx]

**S3 Table**. **Rates of virologic response and virologic failure to paritaprevir/ritonavir/ ombitasvir and dasabuvir ± ribavirin (ITT analysis)**

|  |  | HCV | HIV/HCV | Total |
| --- | --- | --- | --- | --- |
| Total | N | 2022 (100%) | 386 (100%) | 2408 (100%) |
|  | VR EoT | 1965 (97.2%) | 368 (95.3%) | 2333 (96.9%) |
|  | SVR12 | 1961 (97.0%) | 366 (94.8%) | 2327 (96.6%) |
| Non-response | Non-SVR12 | 61 (3.0%) | 20 (5.2%) | 81 (3.4%) |
|  | - Virologic Failure | 28 (1.4%) | 16 (4.1%) | 44 (1.8%) |
|  | - Relapses | 11 (0.5%) | 6 (1.6%) | 17 (0.7%) |
|  | - Breakthroughs | 4 (0.2%) | 1 (0.3%) | 5 (0.2%) |
|  | - Null responders | 13 (0.6%) | 9 (2.3%) | 22 (0.9%) |
|  | - Non Virologic Reasons | 33 (1.6%) | 4 (1.0%) | 37 (1.5%) |
|  | - Not available (HCV UND <10w) | 19 (0.9%) | 1 (0.3%) | 20 (0.8%) |
|  | - Failure to return (loss to follow-up) | 8 (0.4%) | 0 | 8 (0.3%) |
|  | - Other reason | 1 (0,01%) | 1 (0.3%) | 2 (0.1%) |
|  | - Not available (Reason unknown) | 5 (0.2%) | 2 (0.5%) | 7 (0.3%) |

*Abbreviations: HCV (Hepatitis C virus); HIV (Human Immunodeficiency Virus); VR EoT (virologial response end of treatment); SVR12 (sustained virologic response at 12 weeks); ITT (intention-to-treat)*
